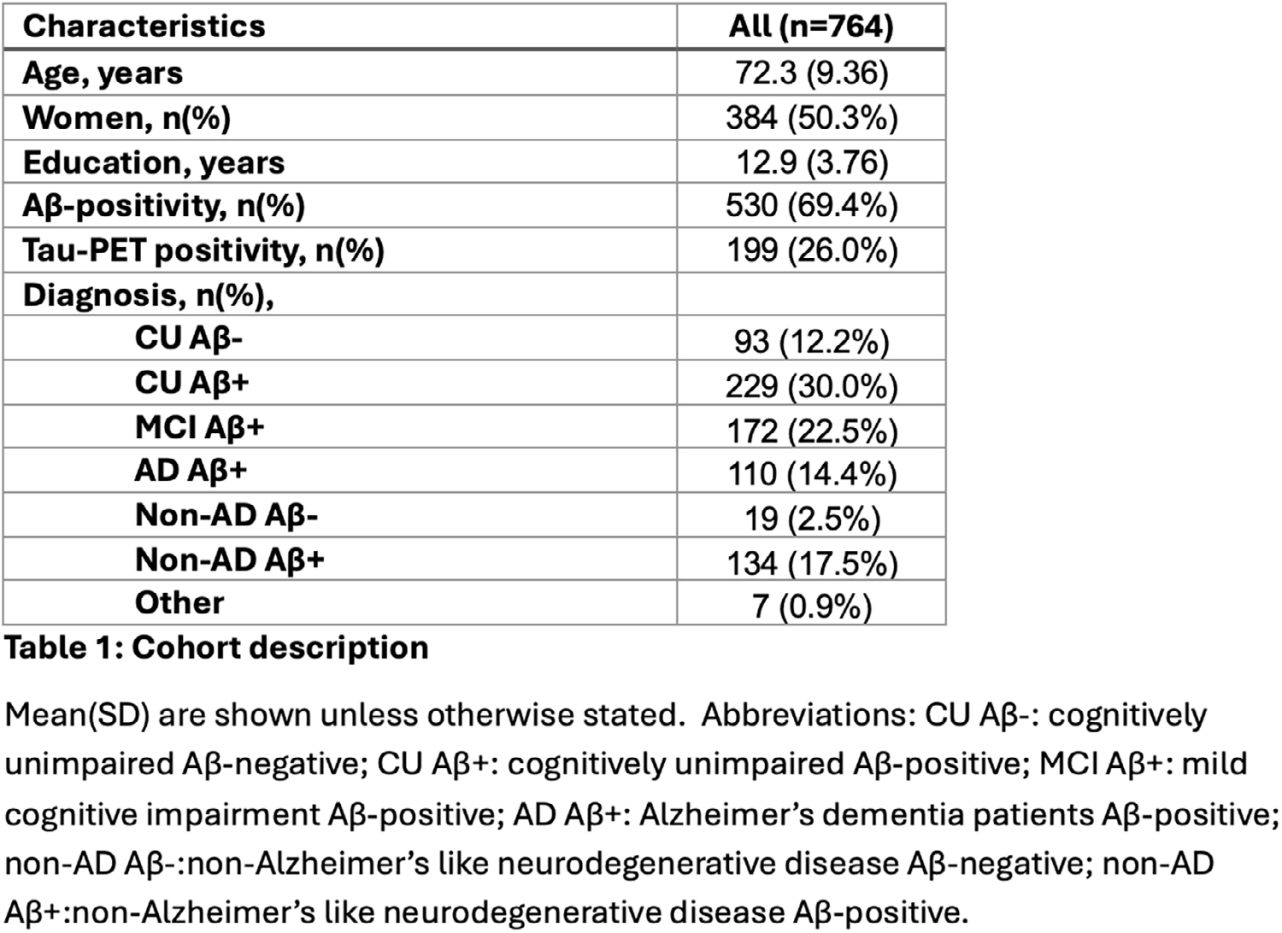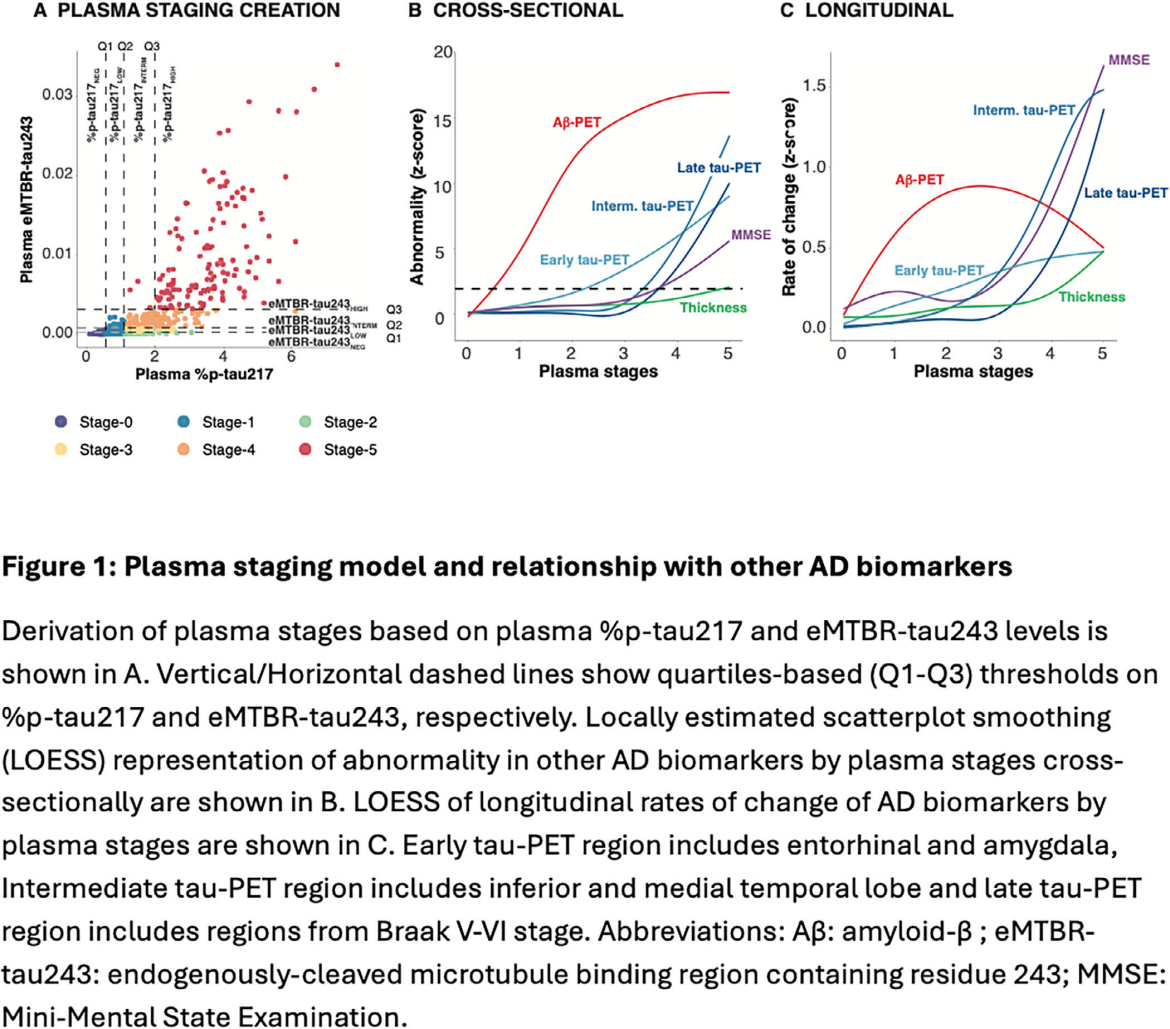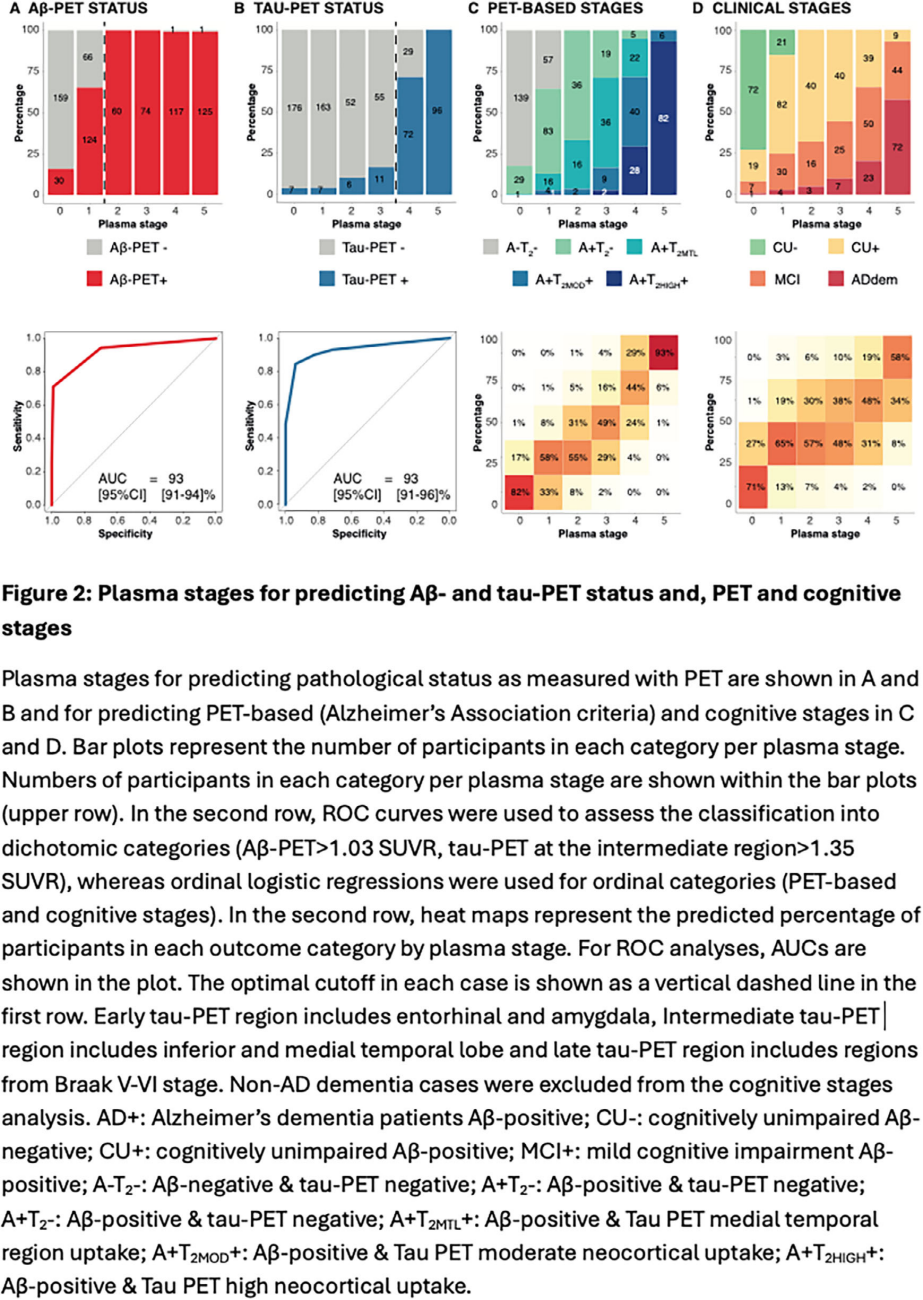# Biological staging of Alzheimer's disease with the novel plasma eMTBR‐tau243 and %p‐tau217

**DOI:** 10.1002/alz70856_098889

**Published:** 2025-12-24

**Authors:** Gemma Salvadó, Kanta Horie, Nicolas R. Barthélemy, Suzanne E. Schindler, Shorena Janelidze, Anna Orduña Dolado, Divya Bali, Erik Stomrud, Niklas Mattsson‐Carlgren, Sebastian Palmqvist, Jacob W. Vogel, Randall J. Bateman, Rik Ossenkoppele, Oskar Hansson

**Affiliations:** ^1^ Clinical Memory Research Unit, Department of Clinical Sciences, Lund University, Lund, Sweden; ^2^ Barcelonaβeta Brain Research Center (BBRC), Pasqual Maragall Foundation, Barcelona, Spain; ^3^ Eisai Co, Tsukuba, Japan; ^4^ Hope Center for Neurological Disorders, Washington University School of Medicine, St. Louis, MO, USA; ^5^ Washington University in St. Louis School of Medicine, St. Louis, MO, USA; ^6^ The Tracy Family SILQ Center, St. Louis, MO, USA; ^7^ Department of Neurology, Washington University in St. Louis School of Medicine, St. Louis, MO, USA; ^8^ Knight Alzheimer Disease Research Center, Washington University School of Medicine, St. Louis, MO, USA; ^9^ Washington University in St. Louis, St. Louis, MO, USA; ^10^ Clinical Memory Research Unit, Department of Clinical Sciences, Lund University, Lund, Skåne, Sweden; ^11^ Memory Clinic, Skåne University Hospital, Malmö, Skåne, Sweden; ^12^ Clinical Memory Research Unit, Department of Clinical Sciences Malmö, Lund University, Lund, Sweden; ^13^ Clinical Memory Research Unit, Lund University, Malmö, Skåne, Sweden; ^14^ Department of Neurology, Skåne University Hospital, Lund, Sweden; ^15^ Wallenberg Center for Molecular Medicine, Lund University, Lund, Sweden; ^16^ Department of Clinical Sciences Malmö, Faculty of Medicine, SciLifeLab, Lund University, Lund, Sweden; ^17^ Knight Alzheimer Disease Research Center, St. Louis, MO, USA; ^18^ Washington University School of Medicine, St. Louis, MO, USA; ^19^ Clinical Memory Research Unit, Department of Clinical Sciences Malmö, Faculty of Medicine, Lund University, Lund, Sweden; ^20^ Amsterdam Neuroscience, Brain Imaging, Amsterdam, Netherlands; ^21^ Alzheimer Center Amsterdam, Neurology, Vrije Universiteit Amsterdam, Amsterdam UMC location VUmc, Amsterdam, Netherlands; ^22^ Memory Clinic, Skåne University Hospital, Malmö, Sweden

## Abstract

**Background:**

Given recent advancements in clinical trials and disease‐modifying treatments, the development of scalable, low‐invasive methods for staging biological Alzheimer's disease (AD) is an urgent need. Current plasma biomarkers lack the specificity required for accurately assessing the advanced stages of AD. This study introduces a staging method based on two plasma high‐performing biomarkers. The plasma %p‐tau217, which is an early marker of amyloid‐β pathology, and the novel plasma biomarker eMTBR‐tau243, referring to endogenously‐cleaved (thus “eMTBR”) microtubule binding region containing residue 243, which reflects aggregated tau pathology.

**Method:**

We included 764 individuals from BioFINDER‐2 covering the AD continuum (Table 1). Plasma %p‐tau217 and eMTBR‐tau243 levels were measured by mass‐spectrometry. Participants were categorized into four quartile‐based biomarker levels (negative, low, intermediate, high) and staged using a combined biomarker system: Stage‐0 (*p*‐tau217_NEG_), Stage‐1 (*p*‐tau217_LOW_), Stage 2 (*p*‐tau217_INTERMED/HIGH_ & eMTBR_NEG_), Stage‐3 (*p*‐tau217_INTERMED/HIGH_ & eMTBR_LOW_), Stage‐4 (*p*‐tau217_INTERM/HIGH_ & eMTBR_INTERMED_), and Stage‐5 ((*p*‐tau217_INTERM/HIGH_ & eMTBR_HIGH;_ Figure 1A). Plasma‐based stages were compared with other AD biomarkers, including amyloid‐β and tau PET, cortical thickness, and cognitive measures, both cross‐sectionally and longitudinally. We also tested the predictive accuracy of plasma‐based stages against amyloid‐β and tau‐PET status, using receiver operating characteristic [ROC] curve, as well as PET‐based (based on the AA revised criteria) and diagnostic stages, using ordinal logistic regression.

**Result:**

Plasma %p‐tau217 levels rose markedly before increases in eMTBR‐tau243, suggesting sequential biomarker dynamics (Figure 1A). Higher plasma biomarker stages were associated with more abnormal imaging and clinical AD features, consistent with the expected disease progression (Figure 1B). Longitudinal changes in these AD features were also aligned with the staging system (Figure 1C). Individuals at higher plasma biomarker stages were more likely to be amyloid‐β‐PET positive (area under the curve [AUC]=0.93[0.91‐0.94]%, threshold: plasma stage 2+) and tau‐PET positive (AUC=0.93[0.91‐96]%, threshold: plasma stage 4+; Figure 2A‐B). Higher plasma stages were also associated with more severe PET‐based (C‐statistic[95%CI]=0.91[0.90‐93]%) and clinical stages (C‐statistic[95%CI]=0.83[0.81‐0.85]%; Figure 2C‐D).

**Conclusion:**

The combination of plasma %p‐tau217 and eMTBR‐tau243 and facilitates accurate biological staging of AD. This plasma‐based approach is non‐invasive, scalable, and offers a more practical alternative to cerebrospinal fluid or PET‐based staging methods.